# Do serum biomarkers really measure breast cancer?

**DOI:** 10.1186/1471-2407-9-164

**Published:** 2009-05-28

**Authors:** Jonathan L Jesneck, Sayan Mukherjee, Zoya Yurkovetsky, Merlise Clyde, Jeffrey R Marks, Anna E Lokshin, Joseph Y Lo

**Affiliations:** 1 Cancer Program, Broad Institute of Harvard University and Massachusetts Institute of Technology, Cambridge, MA 02142, USA; 2 Department of Pediatric Oncology, Dana-Farber Cancer Institute, Boston, MA 02215, USA; 3 Department of Statistical Science, Duke University, Durham, NC 27708, USA; 4 Institute for Genome Sciences and Policy, Duke University, Durham, NC 27708, USA; 5 Department of Computer Science, Duke University, Durham, NC 27708, USA; 6 Division of Hematology/Oncology, University of Pittsburgh School of Medicine, Pittsburgh, PA 15213, USA; 7 University of Pittsburgh Cancer Institute, Pittsburgh, PA 15213, USA; 8 Department of Experimental Surgery, Duke University Medical Center, Durham, North Carolina 27710, USA; 9 Duke Advanced Imaging Labs, Department of Radiology, Duke University Medical Center, Durham, North Carolina 27710, USA; 10 Department of Biomedical Engineering, Duke University, Durham, NC 27708, USA; 11 Medical Physics Graduate Program, Duke University, Durham, NC 27708, USA

## Abstract

**Background:**

Because screening mammography for breast cancer is less effective for premenopausal women, we investigated the feasibility of a diagnostic blood test using serum proteins.

**Methods:**

This study used a set of 98 serum proteins and chose diagnostically relevant subsets via various feature-selection techniques. Because of significant noise in the data set, we applied iterated Bayesian model averaging to account for model selection uncertainty and to improve generalization performance. We assessed generalization performance using leave-one-out cross-validation (LOOCV) and receiver operating characteristic (ROC) curve analysis.

**Results:**

The classifiers were able to distinguish normal tissue from breast cancer with a classification performance of AUC = 0.82 ± 0.04 with the proteins MIF, MMP-9, and MPO. The classifiers distinguished normal tissue from benign lesions similarly at AUC = 0.80 ± 0.05. However, the serum proteins of benign and malignant lesions were indistinguishable (AUC = 0.55 ± 0.06). The classification tasks of normal vs. cancer and normal vs. benign selected the same top feature: MIF, which suggests that the biomarkers indicated inflammatory response rather than cancer.

**Conclusion:**

Overall, the selected serum proteins showed moderate ability for detecting lesions. However, they are probably more indicative of secondary effects such as inflammation rather than specific for malignancy.

## Background

The high prevalence of breast cancer motivates the development of better screening and diagnostic technologies. To complement mammography screening, which has moderate sensitivity [1] and specificity [2-4] and low positive-predictive value in younger women [1], researchers have investigated the efficacy of detecting breast cancer using proteins. Proteins offer detailed information about tissue health conditions, allowing the identification of cancer type and risk, and thereby prompting potentially better-targeted and more effective treatment. Some studies have correlated breast cancer prognosis with proteins in the tumor [5], such as hormone receptors, HER-2, urokinase plasminogen activator, and plasminogen activator inhibitor 1 [6,7], and caPCNA [8]. However, accessing these local proteins requires biopsies, which is not practical for a screening regimen. We therefore consider serum proteins. Serum and plasma protein-based screening tests have already been developed for many diseases, such as Alzheimer's disease [9], cardiovascular disease [10], prostate cancer [11], and ovarian cancer [12].

For breast cancer, however, there are currently very few serum markers used clinically. Some studies have identified as possible breast cancer markers the proteins CA 15.3 [13-15], BR 27.29, tissue polypeptide antigen (TPA), tissue polypeptide specific antigen (TPS), shed HER-2 [15], and BC1, BC2, and BC3 [16,17]. However, other studies found a lack of sufficient diagnostic ability in serum proteins, including CA 15.3 [13,17-20], CA 125 [20], CA 19.9 [20], CA 125 [20], BR 27.29 [13,18], and carcinoembryonic antigen (CEA) [13]. The European Group of Tumor Markers identified the MUC-1 mucin glycoproteins CA 15.3 and BR 27.29 as the best serum markers for breast cancer, but they could not recommend these proteins for diagnosis due to low sensitivity [18].

Cancer biomarkers are valued according to their specificity and sensitivity, suiting them for different clinical roles. For example, general population screening requires high sensitivity but not necessarily high specificity, if a low-cost secondary screen is available. Conversely, disease recurrence monitoring requires high specificity but not necessarily high sensitivity, if a more sensitive secondary test is available. Therefore to optimize clinical utility, it is important to measure the sensitivity and specificity of any proposed biomarker. Because solid tumors cause many changes in the surrounding tissue, it is likely that some potential biomarkers measure the body's response to cancer rather than the cancer itself. Cancer marker levels may be increased due to various secondary factors, such as therapy-mediated response [21-23] and benign diseases. For example, CA 15-3 levels increase in chronic active hepatitis, liver cirrhosis, sarcoidosis [24], hypothyroidism [25], megablastic anemia [26], and beta-thalassaemia [27]. To help measure biomarker specificity in our study, we included normal, benign, and malignant samples.

In general, breast cancer biomarker studies have found individual circulating proteins, but it is important to consider covariation of multiple protein levels. It is useful to know which combinations of proteins may yield high diagnostic performance, even though each protein individually might yield low performance [28,29]. Some studies have detected discriminatory breast cancer biomarkers using mass spectrometry. [16,30-36] Because of mass spectrometry's peak interpretation and reproducibility challenges [37,38], scientists have searched for breast cancer biomarkers from predefined collections of known candidate markers using newer multiplex technologies, such as reverse-phase protein microarray [39]. To our knowledge, ours is the first study to assess a large set of serum proteins collectively by a sensitive and specific multiplex assay in order to identify the most promising proteins for breast cancer detection.

Although studies have shown correlations between serum proteins and breast cancer, it is often unclear how these correlations translate into clinical applicability for diagnosis. To quantify the diagnostic performance of a set of proposed biomarkers, we have implemented many statistical and machine-learning models. We have focused on Bayesian models, which provide full posterior predictive distributions. This detailed information would help a physician to judge how much emphasis to place on the classifier's diagnostic prediction.

The goal of this study was to quantify the ability of serum proteins to detect breast cancer. For measuring predictive performance on this noisy data set, we used many statistical classification models. To better understand the cancer-specificity of the screening test, we ran the classifiers on proteins from malignant lesions, benign lesions, and normal breast tissue. Our data indicated that some serum proteins can detect moderately well the presence of a breast lesion, but they could not distinguish benign from malignant lesions.

## Methods

### Enrolling subjects

This study enrolled 97 subjects undergoing diagnostic biopsy for breast cancer and 68 normal controls at Duke University Medical Center from June 1999 to October 2005. Women donating blood to this study were either undergoing image guided biopsy to diagnose a primary breast lesion (benign and cancer) or were undergoing routine screening mammography and had no evidence of breast abnormalities (normal). All subjects were enrolled only after obtaining written informed consent for this Duke IRB approved study (P.I., JRM, current registry number, 9204-07-11E1ER, Early Detection of Breast Cancer Using Circulating Markers). All subjects were premenopausal women. Table 1 shows the demographics of the study population. Additional file 1 shows subjects' ages and histology findings.

**Table 1:** Subject demographics

	Normal	Benign	Malignant	Total
Number of subjects	68 (41%)	48 (29%)	49 (30%)	165 (100%)

Mean age (years)	36 ± 8	38 ± 9	42 ± 4	38 ± 8

Race: Black	23 (41%)	19 (34%)	14 (25%)	56 (34%)

Race: White	45 (41%)	29 (27%)	35 (32%)	109 (66%)

### Measuring serum protein levels with ELISA

Blood sera were collected under the HIPAA-compliant protocol "Blood and Tissue Bank for the Discovery and Validation of Circulating Breast Cancer Markers." Blood was collected from subjects prior to surgical resection. All specimens were collected in red-stoppered tubes and processed generally within 4 hours (but not greater than 12 hours) after collection and stored at -80°C. Sera were assayed using the Enzyme-Linked ImmunoSorbent Assay (ELISA, Luminex platform) and reagents in the Luminex Core Facility of University of Pittsburgh Cancer Institute. The Luminex protocol was optimized for analytical performance as described by Gorelik *et al*. [12]. One replicate per patient sample was performed with reactions from 100 beads measured and averaged. All samples were analyzed on the same day using the same lot of reagents. Complete information about characteristics of individual assays including inter- and intra-assay coefficients of variation (CVs) is available from the manufacturers of assays [see Additional file 2] and from the Luminex Core website http://www.upci.upmc.edu/luminex/sources.cfm. Based on our analysis of assays performed monthly within one month interval for 3 months using the same lot of reagents, the intra-assay CV for different analytes was in the range of 0.7–11% (typically < 5%) and inter-assay 3.7–19% (<11% for same lot reagents).

Biomarkers were selected based on the known literature reports about their association with breast cancer. The 98 assayed proteins are shown in Table 2, with further details in Additional file 2. In addition to the protein levels, patient age and race were also recorded.

**Table 2:** List of the 98 serum proteins measured by ELISA assay (Luminex platform)

ACTH	Adiponectin	AFP	Angiostatin
Apolipoprotein A1	Apolipoprotein Apo A2	Apolipoprotein Apo B	Apolipoprotein Apo C2

Apolipoprotein Apo C3	Apolipoprotein Apo E	CA 15-3	CA 19-9

CA-125	CA72-4	CD40L (TRAP)	CEA

Cytokeratin 19	DR5	EGF	EGFR

EOTAXIN	ErbB2	FGF-b	Fibrinogen

Fractalkine	FSH	G-CSF	GH

GM-CSF	GROa	Haptoglobin	HGF

IFN-a	IFN-g	IGFBP-1	IL-10

IL-12p40	IL-13	IL-15	IL-17

IL-1a	IL-1b	IL-1Ra	IL-2

IL-2R	IL-4	IL-5	IL-6

IL-7	IL-8	IP-10	Kallikrein 10

Kallikrein 8	Leptin	LH	MCP-1

MCP-2	MCP-3	Mesothelin(IgY)	MICA

MIF	MIG	MIP-1a	MIP-1b

MMP-1	MMP-12	MMP-13	MMP-2

MMP-3	MMP-7	MMP-8	MMP-9

MPO	NGF	PAI-I(active)	PROLACTIN

RANTES	Resistin	S-100	SAA

SCC	sE-Selectin	sFas	sFasL

sICAM-1	sIL-6R	sVCAM-1	TGFa

TNF-a	TNF-RI	TNF-RII	tPAI-1

TSH	TTR	ULBP-1	ULBP-2

ULBP-3	VEGF		

### Regression with variable selection

In order to incorporate these proteins into a breast cancer screening tool, we built statistical models linking the protein levels to the probability of malignancy. We used the following three common regression models: linear regression *Y*_*i*_= *X*_*i*_*β *+*ε*_*i*_, *ε *~*N*(0, *σ*^2^), logistic regression , and probit regression Pr(*Y*_*i *_= 1|*β*) = Φ(*X*_*i*_, *β*), where *Y *is the response vector (breast cancer diagnosis), *X *is the matrix of observed data (protein levels), *β *is the vector of coefficients, *ε *is additive noise, and Φ(·) is the cumulative distribution function of the normal distribution. These classical models become unstable and predict poorly if there are relatively few observations (curse of dataset sparsity [40]) and many features (curse of dimensionality [41]). It is better to choose a subset of useful features. We used stepwise feature selection [42-46] to choose the set of proteins that optimized model fit.

However, choosing only one feature subset for prediction comes with an inherent risk. When multiple possible statistical models fit the observed data similarly well, it is risky to make inferences and predictions based only on a single model [47]. In this case predictive performance suffers, because standard statistical inference typically ignores model uncertainty.

### Accounting for model uncertainty with Bayesian model averaging

We accounted for model-selection ambiguity by using a Bayesian approach to average over the possible models. We considered a set of models *M*_1_,..., *M*_*B*_, where each model *M*_*k *_consists of a family of distributions p(*D*|*θ*_*k*_, *M*_*k*_) indexed by the parameter vector *θ*_*k*_, where *D *= (*X*, *Y*) is the observed data. *Y *is the response vector (breast cancer diagnosis), and *X *is the matrix of observed data (protein levels). Using a Bayesian method to average over the set of considered models [47-52], we first assigned a prior probability distribution p(*D*|*M*_*k*_) to the parameters of each model *M*_*k*_. This formulation allows a conditional factorization of the joint distribution,(1)

Splitting the joint distribution in this way allowed us to implicitly embed the various models inside one large hierarchical mixture model. This form allowed us to fit these models using the computational machinery of Bayesian model averaging (BMA).

BMA accounts for model uncertainty by averaging over the posterior distributions of multiple models, allowing for more robust predictive performance. If we are interested in predicting a future observation *D*_*f *_from the same process that generated the observed data *D*, then we can represent the predictive posterior distribution p(*D*_*f*_|*D*) as an average over the models, weighted by their posterior probabilities [47,53,54]:(2)

where the sum's first term p(*D*_*f*_|*D*, *M*_*k*_) is a posterior weighted mixture of conditional predictive distributions(3)

and the sum's second term p(*M*_*k*_|*D*) is a model's posterior distribution(4)

which incorporates the model's marginal likelihood(5)

### Promoting computational efficiency with iterated BMA

BMA allows us to average over all possible models, containing all possible subsets of features. However, considering many models would require extensive computations, especially when computing the posterior predictive distributions. Such computations would be prohibitively long for a quick screening tool that is intended not to impede clinicians' workflow during routine breast cancer screening. Because it was computationally infeasible to consider all possible 2^100^≈1.26*10^30 ^models, we first chose a set of the best fitting models. For computational efficiency in model selection, this study followed Yeung *et al*. [55] and used a deterministic search based on an Occam's window approach [54] and the "leaps and bounds" algorithm [56] to identify models with higher posterior probabilities.

In addition to choosing the best models, we also chose the best proteins. We applied an iterative adaptation of BMA [55]. This method initially ranks each feature separately by the ratio of between-group to within-group sum of squares (BSS/WSS) [57]. For protein *j *the ratio is(6)

where I(·) is an indicator function, *X*_*ij *_is the level of protein *j *in sample *i*,  and  are respectively the average levels of protein *j *in the normal and cancer groups, and  is the average level of protein *j *over all samples.

Ordered by this BSS/WSS ranking, the features were iteratively fit into BMA models, which generated posterior probability distributions for the proteins' coefficients. We then discarded proteins that had low posterior probabilities of relevance, Pr(*b*_*j *_≠ 0| *D*) < 1%, where(7)

where Pr(*b*_*j *_≠ 0| *D*) is the posterior probability that protein j's coefficient is nonzero, Γ is the subset of the considered models *M*_1_,..., *M*_*B *_that include protein *j*. By discarding proteins that have small influence on classification, this iterative procedure keeps only the most relevant proteins.

### Other models for high-dimensional data

To compare iterated BMA's classification and generalization performance, we also classified the data using two other dimensionality-reducing methods: a support vector machine (SVM) [58] with recursive feature selection [59,60] and least-angle regression (LAR, a development of LASSO) [61].

All modeling was performed using the R statistical software (version 2.6.2), and specifically the BMA package (version 3.0.3) for iterated BMA, the packages e1071 (version 1.5–16) and R-SVM [31] for the SVM with recursive feature selection, the lars package (version 0.9–5) for least angle regression, and the ROCR package (version 1.0–2) for ROC analysis. We extended the BMA package to compute the full predictive distributions (See Equations 2–6) within cross-validation using an MCMC approach. Additional file 3 contains the R code and Additional file 4 contains the data in comma-delimited format.

### Evaluating classification performance

The classifiers' performances were analyzed and compared using receiver operating characteristic (ROC) analysis [62]. ROC curve metrics were compared statistically using a nonparametric bootstrap method [63]. To estimate generalization performance on future cases, we first defined independent train and test sets by randomly choosing 70% of samples for training and optimizing the classifiers, and then we tested the classifiers on the remaining 30% of the samples. To compare with the train/test split, we also performed leave-one-out cross-validation (LOOCV). Feature selection was performed within each fold of the cross-validation.

### Evaluating feature-Selection methods

We compared models with feature-selection techniques using two methods: feature concentration and classifier performance. For feature concentration, we performed feature selection within each fold of a LOOCV. We recorded how many times each feature was chosen. This method distinguished the feature selection methods that chose few versus many features. Using classifier performance, we investigated the effect of feature selection and sampling. We compared linear models run on the data with these four techniques: 1) no feature selection (using all the proteins in the model), 2) preselecting the features (using all the data to choose the best features, and then running the model using only those preselected features in LOOCV), 3) stepwise feature selection, and 4) iterated BMA. For each case, we ran the classifier in LOOCV.

## Results

### Classifier performance

**Figure 1:**
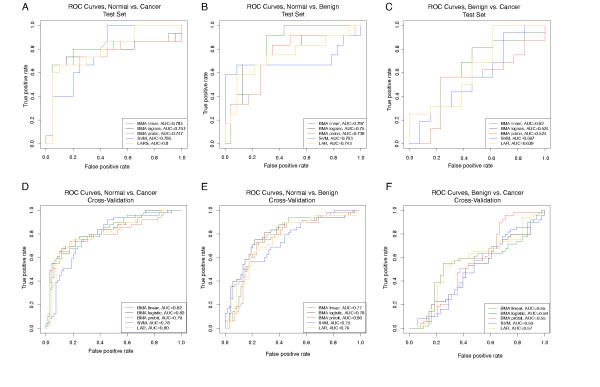
**ROC curves showing the classification performance of statistical models using the serum protein levels**. The models were run with a 70% train and 30% test split of the data set (A-C) and also with leave-one-out cross-validation (LOOCV) (D-F). The classifiers performed similarly, with moderate classification results for normal vs. malignant or benign lesions (A, B, D, E) and poor classification results for malignant vs. benign lesions (C, F).

The classifiers achieved moderate classification performance for both normal vs. malignant (*AUC *= 0.77 ± 0.02 on the test set, and *AUC *= 0.80 ± 0.02 for LOOCV) and normal vs. benign (*AUC *= 0.75 ± 0.03 on the test set, and *AUC *= 0.77 ± 0.02 for LOOCV), but very poor performance for malignant vs. benign tumors (*AUC *= 0.57 ± 0.05 on the test set, and *AUC *= 0.53 ± 0.03 for LOOCV). The classification performance is shown as ROC curves in Figure 1. Whereas the ROC curves show the classification performance over the entire range of prediction thresholds, we also considered the threshold of Pr(*Y*_*i *_= 1|*β*) = 0.5 in particular. For this threshold, Table 3 shows the classification error. The models performed similarly, with approximately 20 false negatives and 10 false positives. All classifiers were run with leave-one-out cross-validation (LOOCV). The classifiers chose the best subsets of proteins for classification.

**Table 3:** Cross-validation classification errors, normal versus cancer

Model	FN	FP
BMA of linear models	19	8

BMA of logistic models	15	12

BMA of probit models	18	7

SVM with RFS	18	12

LAR	24	5

**Figure 2:**
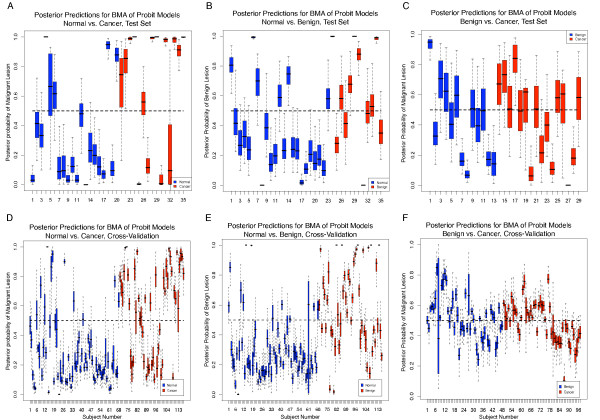
**Posterior predictions of Bayesian model averaging (BMA) of probit models, run with a 70% train and 30% test split of the data set (A-C) and also with leave-one-out cross-validation (LOOCV) (D-F)**. The classifiers achieved moderate classification results for normal vs. malignant or benign lesions (A, B, D, E) and poor classification results for malignant vs. benign lesions (C, F).

Figure 2 plots the full posterior predictive distributions for BMA of probit models run with LOOCV. In general, the predictive distributions were more "decided" (concentrated further from the 0.5 probability line) for the tasks of normal vs. cancer and normal vs. benign, but they were less "decided" (concentrated closer to the 0.5 probability line) for benign vs. cancer. This trend indicated that the serum protein levels were very similar for malignant and benign lesions.

### Selected serum proteins

**Figure 3:**
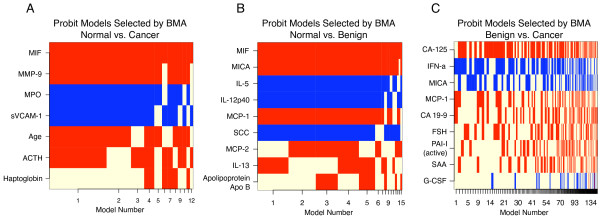
**Models selected by BMA of linear models**. Features are plotted in decreasing posterior probability of being nonzero. Models are ordered by selection frequency, with the best, most frequently selected models on the left and the weakest, rarest chosen on the right. Coefficients with positive values are shown in red and negative values in blue. Strong, frequently selected features appear as solid horizontal stripes. A beige value indicates that the protein was not selected in a particular model.

The iterated BMA algorithm chose the best-fitting probit models. The chosen models and their proteins are shown in Figure 3. The best proteins for each classification task are listed in Table 4. The top protein for both normal vs. cancer and normal vs. benign was macrophage migration inhibitory factor (MIF), a known inflammatory agent [64-66]. Other selected proteins also play roles in inflammation and immune response, such as MMP-9 [67,68], MPO [69], sVCAM-1 [70], ACTH [71], MICA [72], IL-5 [73], IL-12 p40 [74-76], MCP-1 [77], and IFNa [78-80]. For benign vs. cancer, the top protein was CA-125, which is used as a biomarker for ovarian cancer [12,81-83]. However, the greater presence of CA-125 in cancer tissue was still too subtle to allow the classifiers to achieve good classification performance.

**Table 4:** Proteins chosen by BMA of linear models

Protein	Biological Role	Higher Prevalence In
*Normal vs. Cancer*		

MIF (macrophage migration inhibitory factor)	Inflammation, regulates macrophage function in host defense through the suppression of anti-inflammatory effects of glucocorticoids [64-66]	Cancer

MMP-9 (matrix metalloproteinase)	Breakdown and remodeling of extracellular matrix [67], regulates growth plate angiogenesis and apoptosis of hypertrophic chondrocytes [68]	Cancer

MPO (myeloperoxidase)	Inflammation, produces HOCl, modulates vascular signaling and vasodilatory functions of nitric oxide (NO) during acute inflammation [69]	Normal

sVCAM-1 (soluble vascular cell adhesion molecule 1)	Mediates leukocyte-endothelial cell adhesion and signal transduction, membrane-bound adhesion molecules and the process of vascular inflammation of the vessel wall [70]	Normal

ACTH (adrenocorticotropic hormone)	Stimulates secretion of adrenal corticosteroids [71]	Cancer

*Normal vs. Benign*		

MIF (macrophage migration inhibitory factor)	Inflammation, regulates macrophage function in host defense through the suppression of anti-inflammatory effects of glucocorticoids [64-66]	Benign

MICA (MHC class I polypeptide-related sequence A)	Stress-induced antigen that is broadly recognized by intestinal epithelial gamma delta T cells, ligands for natural killer cells [72]	Benign

IL-5 (Interleukin 5)	Stimulates B cell growth, increases immunoglobulin secretion, mediates eosinophil activation [73]	Normal

IL-12 p40 (Interleukin 12, p40 chain)	Differentiation of naive T cells into Th1 cells, stimulates the growth and function of T cells, stimulates the production of interferon-gamma (IFN-γ) and [74,75](TNF-α) from T and natural killer cells, and reduces IL-4 mediated suppression of IFN-γ [76]	Normal

MCP-1 (Monocyte chemotactic protein-1)	Induces recruitment of monocytes, T lymphocytes, eosinophils, and basophils and is responsible for many inflammatory reactions to disease [77]	Benign

*Benign vs. Cancer*		

CA-125 (cancer antigen 125)	Marker for ovarian cancer [12,81,82]	Cancer

IFNa (Interferon type I)	Secreted by leukocytes, fibroblasts, or lymphoblasts in response to viruses [78] or interferon inducers; implicated in autoimmune diseases [79,80]	Benign

MICA (MHC class I polypeptide-related sequence A)	Stress-induced antigen that is broadly recognized by intestinal epithelial gamma delta T cells, ligands for natural killer cells [72]	Benign

**Figure 4:**
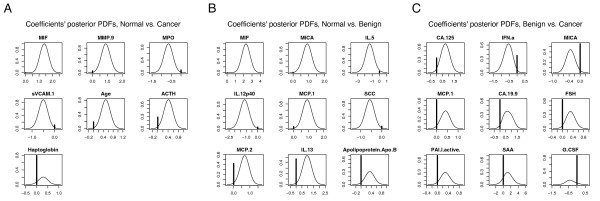
**Posterior distributions of the model coefficients for the proteins**. The distributions are mixtures of a point mass at zero and a normal distribution. The height of the solid line at zero represents the posterior probability that the coefficient is zero. The nonzero part of the distribution is scaled so that the maximum height is equal to the probability that the coefficient is nonzero.

Complementary to the models' matrix plots for feature strength are the coefficients' marginal posterior probability distribution functions (PDFs), which the BMA technique calculates by including information from all considered models. Figure 4 shows the marginal posterior PDFs for the top coefficients for the BMA models. The coefficients' distributions are mixture models of a normal distribution and a point mass at zero. This point mass is much larger for the benign vs. cancer models than for normal vs. cancer and normal vs. benign models. The higher weight at zero indicates that the proteins are less suitable for distinguishing benign from malignant lesions than they are for distinguishing lesions from normal tissue.

### Feature selection and Bayesian model averaging

**Figure 5:**
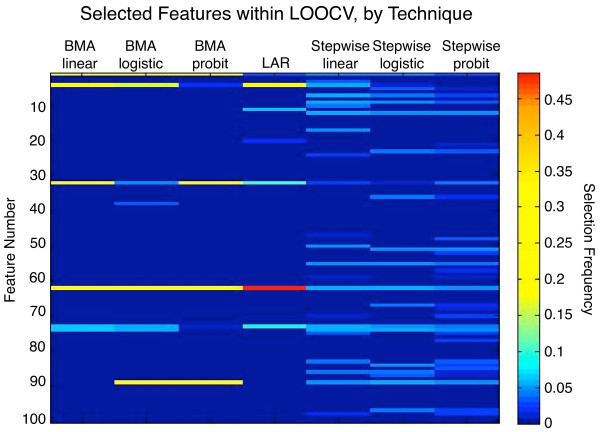
**Heatmap of normalized frequencies of selected features, normal vs. cancer**. The feature selection frequencies were averaged over all folds of the LOOCV. For comparison across techniques, the frequencies in each column were scaled to sum to one. Less-frequently selected features appear as cooler dark blue colors, whereas more frequently selected features appear as hotter, brighter colors. Models that used fewer features appear as dark columns with a few bright bands, whereas models that used more features appear as denser smears of darker bands.

While doing feature selection for normal vs. cancer within LOOCV, we recorded the counts of how many times each protein was selected. The selection frequencies are shown as a heatmap in Figure 5. Iterated BMA and least-angle regression selected the fewest proteins, whereas stepwise feature selection chose many more proteins. The strongest proteins were chosen consistently across all feature selection techniques, as shown by the horizontal lines in the figure.

**Figure 6:**
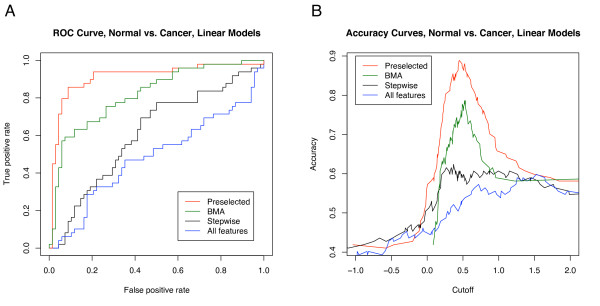
**ROC and accuracy curves for linear models with four feature selection techniques**. 1) Preselected: the features (using all the data to choose the best features, and then running the model using only those preselected features in LOOCV), 2) BMA: iterated Bayesian model averaging, 3) Stepwise feature selection, and 4) All features: using all the proteins in the model, no feature selection.

We also investigated the effect of feature selection upon classifier generalization. Figure 6 shows the ROC and accuracy curves for linear models with various feature selection strategies. Preselecting the features generated a very optimistically biased classification performance. When the same feature selection technique (stepwise) was applied within each fold of LOOCV, the classification performance fell dramatically. Using no feature selection (using all proteins) had extremely poor classification performance – no better than guessing. The poor performance demonstrated that the linear model needs feature selection for good classification performance when the number of features is roughly the same as the number of samples in noisy data. Iterated BMA of linear models significantly outperformed the stepwise, single-model method. This performance increase demonstrated the better predictive ability of the BMA models; averaging a set of the most promising models was better than using only the single best model.

## Discussion

The serum proteins allowed the classifiers to detect lesions (malignant or benign) from normal tissue moderately well, but they were very poor for distinguishing benign from malignant lesions. These classification results were consistent in both the test set and the leave-one-out cross-validation. This consistency implies that the classification results are not highly dependent on the sampling scheme but rather highlight consistent trends in our data set. The classification results show that the proteins were not specific for cancer and suggest that they may indicate states of biological or immunological stress. A good candidate for the dominating biological effect is inflammation, since the top protein selected for both normal vs. cancer and normal vs. benign was macrophage migration inhibitory factor (MIF), which is active in inflammation [64-66]. The best protein for distinguishing malignant from benign tumors was cancer antigen 125 (CA-125), which is a prominent serum marker for ovarian cancer [12,81,83]. However, CA-125 levels are influenced by other factors, such as age, hormone-replacement therapy, smoking habits, and use of oral contraceptives [84]. In general it is very difficult to ascertain whether biomarker proteins are generated by the cancer itself or by secondary effects, such as immune response. Once potential biomarker proteins are identified in initial studies, follow-up studies can focus on those proteins to discover their origin and role in the disease process. Helpful experimental study designs would control for known secondary causes of biomarker activity and would collect enough samples to average over unintended secondary causes. Longitudinal studies would also lessen the effect of transient secondary causes.

To quantify and compare classification performances, we used ROC analysis, which fairly compares classifiers that may be operating at different sensitivities due to arbitrary decision thresholds applied to the classifiers' output values. Although our data set comprised three classes (normal, benign, and cancer), current ROC methods required us to split an inherently three-class classification problem into three different two-class tasks: normal vs. benign, normal vs. cancer, and benign vs. cancer. The field of ROC analysis is still in development for the three-class problem; no consensus has yet been reached about how to quantitatively score the resulting six-dimensional ROC hypersurface [85-88]. However, for other methods of classifier comparison, such as the generalized Brier score or discrete counts of classification errors, full three-class models could have been used, albeit with decision thresholds.

This study investigated a group of 98 serum proteins (Table 2), which is relatively small sample of all detectable serum proteins. Future studies may identify other proteins with stronger relationships to breast cancer. Rather than relying on only a few proteins, upcoming protein technologies will allow the screening of large populations with protein-based tests that require a larger set of proteins. Microfluidics chips would simplify the process of automating blood tests in a high-throughput fashion. However, with current assay technology and cost-benefit analysis of screening programs, the fixed cost per protein assayed essentially limits the number of proteins that can be used for screening. To lower screening costs, we chose small subsets of the features via feature-selection methods. Iterated BMA and least-angle regression were able to classify well using a far smaller set of features than those chosen by stepwise feature selection.

High feature correlation impedes many feature-selection techniques. For stochastic feature-selection methods, two highly correlated features are each likely to be chosen in alternation. Similarly, a cluster of highly correlated features causes the feature selection technique to spread the feature selection rate among each feature in the cluster, essentially diluting each feature's selection rate. Severe dilution of selection rates can cause none of the cluster's features to be selected. Future work will entail adding cluster-based methods to the iterated BMA algorithm.

The currently proposed serum biomarkers for breast cancer are not sensitive or specific enough for breast cancer screening. However, better biomarkers may be identified with newer protein assay technology and larger data sets. A protein's subtle diagnostic ability may be enhanced by the assimilation of other medical information, such as gene expression and medical imaging. The proteins will boost diagnostic performance only if they provide complementary and non-redundant information with the clinical practice of mammograms, sonograms, and physical examination. The relationship of medical imaging and protein screening remains to be determined in future work.

## Conclusion

We have performed feature-selection and classification techniques to identify blood serum proteins that are indicative of breast cancer in premenopausal women. The best features to detect breast cancer were MIF, MMP-9, and MPO. While the proteins could distinguish normal tissue from cancer and normal tissue from benign lesions, they could not distinguish benign from malignant lesions. Since the same protein (MIF) was chosen for both normal vs. cancer and normal vs. benign lesions, it is likely that this protein plays a role in the inflammatory response to a lesion, whether benign or malignant, rather than in a role specific for cancer. While the current set of proteins show moderate ability for detecting breast cancer, their true usefulness in a screening program remains to be seen in their integration with imaging-based screening practices.

## List of abbreviations used

This article used the following abbreviations: AUC: for Area under the ROC curve; BMA: for Bayesian model averaging; CV: for coefficient of variation; ELISA: for enzyme-linked immunosorbent assay; HIPAA: for Health Insurance Portability and Accountability Act; LOOCV: for leave-one-out cross-validation; MICA: for human major histocompatibility complex class I chain-related A; MIF: for macrophage migration inhibitory factor; MMP-9: for matrix metalloproteinase 9; PDF: for probability density function; and ROC: for receiver operating characteristic.

## Competing interests

The authors declare that they have no competing interests.

## Authors' contributions

JRM coordinated the sample collection and study design. AEL and ZY conducted the ELISA assays. JLJ performed the computations and statistical analysis. SM and MC guided the use of Bayesian Model Averaging in the study. JLJ and JYL contributed to the drafting of the manuscript, which was approved by all authors.

## Pre-publication history

The pre-publication history for this paper can be accessed here:

http://www.biomedcentral.com/1471-2407/9/164/prepub

## Supplementary Material

Additional File 1 **Subject diagnoses and protein levels**. These data respresent this study's selected serum proteins and their antibodies. Click here for file

Additional File 2 **Proteins and antibodies**. These data show sample information with the serum protein levels measured by ELISA. Click here for file

Additional File 3 **Classification code in R**. This file is a compressed folder containing this study's source code for the statistical program R. Click here for file

Additional File 4 **Serum protein data**. This file is a comma-delimited file of blood serum ELISA data intended for computational analysis in the R scripts of Additional file 1. Click here for file
